# Dietary Hydroxytyrosol Supplementation on Growth Performance, Gut Morphometry, and Oxidative and Inflammatory Status in LPS-Challenged Broilers

**DOI:** 10.3390/ani14060871

**Published:** 2024-03-12

**Authors:** Kelly M. M. Dias, Carlos H. Oliveira, Arele A. Calderano, Horacio S. Rostagno, Kaique M. Gomes, Kevin E. O’Connor, Reeta Davis, Meg Walsh, James Britton, Enrico A. Altieri, Luiz F. T. Albino

**Affiliations:** 1 Department of Animal Science, Federal University of Viçosa, Viçosa 36570-900, Minas Gerais, Brazil; carlos.oliveira2@ufv.br (C.H.O.); calderano@ufv.br (A.A.C.); rostagno@ufv.br (H.S.R.); kaique.gomes@ufv.br (K.M.G.); 2 Nova Mentis Limited, Nova UCD, Belfield Innovation Park, University College Dublin, Belfield, D04 F438 Dublin, Ireland; koconnor@novamentis.eu (K.E.O.); rdavis@novamentis.eu (R.D.); mwalsh@novamentis.eu (M.W.); jbritton@novamentis.eu (J.B.); ealtieri@novamentis.eu (E.A.A.)

**Keywords:** antioxidant, feed additives, gene expression, jejunum, lipopolysaccharide

## Abstract

**Simple Summary:**

Broiler chickens are frequently exposed to a variety of stress factors in the poultry industry. To simulate these factors, lipopolysaccharide (LPS) from *E. coli* is commonly used as a research model to stimulate inflammatory and oxidative stresses in broilers. LPS can reduce growth performance parameters by negatively affecting broilers’ immune response to inflammation. Feed additives that could mitigate those detrimental effects are often demanded. The present study evaluated the impact of hydroxytyrosol supplementation on the growth performance, gut morphometry, and anti-inflammatory and antioxidant parameters of broilers challenged with LPS. Hydroxytyrosol improved the growth performance, gut morphometry, and oxidative status of broilers challenged with LPS. Therefore, hydroxytyrosol supplementation is a powerful nutritional intervention for mitigating the detrimental effects of LPS.

**Abstract:**

This study assessed the effects of hydroxytyrosol (HT) on 8- to 20-day-old broilers challenged with lipopolysaccharide (LPS); 180 Cobb500™ male chicks were randomly assigned to 3 treatment groups, each comprising 10 replicates with 6 birds per replicate. Treatments included a control diet (CON), CON with LPS administration, and CON + LPS supplemented with 10 mg of HT/kg of feed. LPS was administered intraperitoneally on days 14, 16, 18, and 20. Body weight (BW), body weight gain (BWG), and the feed conversion ratio (FCR) were measured. On day 20, ten birds per treatment were slaughtered for analysis. Bursa, spleen, and liver were collected, and their respective relative weight was determined. The jejunum was destined for morphological analyses of villus height (VH), crypt depth (CD), and their ratio (VH:CD), and for mRNA expression of nuclear factor kappa B (NF-κB), catalase (CAT), glutathione peroxidase (GPx), superoxide dismutase (SOD), and interleukins 10 (IL-10), 1 beta (IL-1β), and 8 (IL-8). HT improved BW, BWG, and FCR, and reduced crypt depth (CD) while increasing the VH:CD ratio in the jejunum. Moreover, HT downregulated mRNA expression of CAT, GPx, IL-10, and IL-1β. In conclusion, HT enhances broiler growth performance, mitigates jejunal mucosa damage from LPS, and modulates antioxidant and immune responses.

## 1. Introduction

In the poultry industry, broilers are frequently exposed to a variety of stress situations such as microbially contaminated feed, pathogen infection, and immunological and oxidative stresses contributing to intestinal mucosa injury and reduction of growth performance [1,2,3]. Lipopolysaccharide (LPS), an integral component of the cell wall found in Gram-negative bacteria, serves as a standard inducer of inflammatory and oxidative stresses in broilers [4,5]. LPS-challenged broilers present an acute inflammatory response and oxidative damage, such as a reduction in the expression and activity of antioxidant enzymes, elevated production of pro-inflammatory cytokines, increased weight of lymphoid organs, and impaired digestion and absorption of nutrients due to morphological damage in the intestine [1,3,6,7].

Plant polyphenols such as curcumin, resveratrol, and hydroxytyrosol (HT) have potential health effects. They are considered potent immunomodulators with many physiological effects due to their antioxidant and anti-inflammatory activities, including for poultry health [8,9,10,11]. HT is found in olives and has been shown to confer many health benefits [8]. It is one of the most powerful naturally known antioxidants and its antioxidant capacity can be attributed to its ortho-dihydroxy configuration within its aromatic ring. According to Bertelli et al. [12], HT’s antioxidant capacity surpasses green tea by tenfold and coenzyme Q10 by twice the amount. Moreover, HT is acknowledged for its role as a scavenger of reactive oxygen species (ROS). HT and its metabolites can reduce the intracellular and extracellular accumulation of ROS and protect vascular endothelial cells from hydrogen peroxide [13,14,15]. Other beneficial effects of HT include the promotion of immunity, anti-inflammatory effects, and the promotion of gut health [7,12,16,17], but previous studies with HT have not shown an increase in broiler growth performance [18,19,20]. 

No studies have been carried out on chickens exposed to LPS and HT. Consequently, there is a lack of knowledge and understanding of how HT influences the immune response, intestinal health, and growth performance of LPS-challenged broiler chickens. We hypothesized that HT has an anti-inflammatory effect, and its dietary supplementation could mitigate the detrimental effects of LPS challenge on broilers’ growth performance. Therefore, this research aimed to evaluate the effects of HT on immune and antioxidant responses, as well as intestinal mucosa regulation through measurement of growth performance, morphological changes in the jejunum, and gene expression of antioxidant enzymes and inflammatory parameters. 

## 2. Materials and Methods

### 2.1. Animals, Experimental Design, and Diets

The study was conducted at the Unit of Study and Research in Poultry Production and Nutrition of the Department of Animal Science, Federal University of Viçosa, Viçosa, Minas Gerais, Brazil.

Cobb500™ male broiler chicks were purchased from a commercial hatchery (Rivelli Alimentos SA, Matheus Leme, MG, Brazil). At the hatchery, all chicks were vaccinated against Marek and Newcastle diseases and infectious bronchitis. Before reaching 8 d, broiler chicks were reared in accordance with the guidelines outlined by Cobb500^®^. During this period, they had unrestricted access to water and a diet formulated with corn and soybean meal under the guidelines established by Rostagno et al. [21].

Following, 180 Cobb500™ male chicks (230.9 g ± 3.44 g) were accommodated in metallic cages (600 cm^2^/bird) and assigned randomly to 3 treatment groups with 10 replicates of 6 birds each, from 8 to 20 days old. Every cage was equipped with a nipple drinker and a trough feeder, ensuring unrestricted access to water and feed throughout the experimental period. The temperature was maintained at 28 °C at the beginning of the experiment, and it was gradually reduced to 22 °C by 20 days of age. Birds were daily exposed to 18 h of continuous light during the experimental period.

The treatments consisted of a control diet (CON), a CON with LPS administration (CON + LPS), and a CON supplemented with 10 mg of hydroxytyrosol/kg of feed with LPS administration (HT + LPS). The experimental diets were formulated based on corn and soybean meal to fulfill the nutritional requirements of the birds according to the guidelines provided by Rostagno et al. [21], without the addition of antibiotics or anticoccidial (Table 1). 1-HT^®^ hydroxytyrosol (>98% purity) used in this study was biologically manufactured and provided by Nova Mentis Ltd., Dublin, Ireland.

*Escherichia coli* LPS (serotype O55:B5, Sigma Chemical Co., St. Louis, MO, USA) was reconstituted in saline solution at a concentration of 1.0 mg/mL and administered to the birds intraperitoneally at a dose of 1 mL/kg of body weight on days 14, 16, 18, and 20. Birds from the control group received the same amount of saline solution (0.9% sodium chloride) via intraperitoneal to guarantee similar handling and stress conditions induced in the ones that received LPS administration.

### 2.2. Growth Performance

At the start and end of the experimental period, which occurred at 8 and 20 days, respectively, the birds and their feed were weighed. Data were collected to measure body weight (BW, kg/bird), BW gain (BWG, kg/bird), feed intake (FI, kg/bird), and feed conversion ratio (FCR, kg/kg). In cases of mortality, the remaining feed was weighed to adjust the FI measurement.

### 2.3. Samples Collection

At 20 days old, one bird with BW closest to the average weight of the experimental unit was chosen for sample collection (totaling 10 birds per treatment). Four hours after the first LPS application, the bird was slaughtered by cervical dislocation for sample collection. Jejunum was identified using Meckel’s diverticulum as a reference, and aseptically removed and separated into two different samples to evaluate intestinal morphometry and determine mRNA content. Furthermore, the liver, spleen, and bursa were removed to determine their relative weights.

#### 2.3.1. Relative Organ Weights

Before slaughtering, the birds selected were weighed. After slaughtering and evisceration, the liver, spleen, and bursa were removed and weighed. Relative organ weight (ROW) was calculated in relation to the birds’ live weight.

#### 2.3.2. Intestinal Morphometry

Samples of the jejunum (≈3 cm length) were obtained and rinsed using a sterile saline solution (0.9% sodium chloride) to clear any luminal contents. The segments were longitudinally incised and affixed to the cardboard using staples, where the serosa remained in contact with the cardboard to not distend or harm its structures [22]. Samples were labeled and preserved in sterile plastic containers filled with a 10% buffered formalin solution. Afterward, the samples underwent dehydration by immersing them in a series of increasing alcohol concentrations, followed by clarification in xylene, and finally embedding in liquid paraffin at 60 °C. The segments were submitted to microtomy to produce semi-serial cross-sections with a thickness of 5 µm. These sections were then stained with hematoxylin and eosin, following Luna’s method [23]. Five 5 µm thick cross-sections were placed on every microscope slide. The slides were observed and photographed using an optical microscope (EVOS^®^ XL Core) with 10× magnification. Subsequently, measurements of villus heights (VH), crypt depths (CD), and the villus height to crypt depth ratio (VH:CD) were performed using ImageJ 1.50i Software, java1.6.0_20 (National Institutes of Health, Bethesda, MD, USA). A total of 20 villi and their crypt were measured for each experimental unit (200 villi and crypt per treatment).

#### 2.3.3. mRNA Extraction and Gene Expression

A second sterile portion of jejunum was obtained, individually stored in cryogenic tubes, and preserved in liquid nitrogen until further analysis. Total RNA extraction was conducted by employing Trizol^®^ (Invitrogen, Carlsbad, CA, USA) following the manufacturer’s instructions. RNase-free silica membrane columns (PureLink™ RNA Mini Kit—Invitrogen TM) were used to wash and isolate the RNA. The resulting precipitate was rehydrated using 30 µL of UltraPure^®^ DNase/RNase-Free water. The RNA concentration was estimated using a NanoDrop™ Lite spectrophotometer (Thermo Fisher Scientific, Beverly, MA, USA) with purity verified by observing A260/A280 ratios within the range of 1.8 to 2.0. RNA integrity was assessed on a 1% agarose gel. Subsequently, the samples underwent DNase treatment and were reverse transcribed into cDNA using the High-Capacity cDNA Reverse Transcription Kits (Applied Biosystems, Thermo Fisher Scientific, Beverly, MA, USA) according to the manufacturer’s instructions.

The target genes evaluated in jejunum were nuclear factor kappa B (NF-κB), catalase (CAT), glutathione peroxidase (GPx), superoxide dismutase (SOD1), and interleukins 1β (IL-1β), 10 (IL-10), and 8 (IL-8). β-actin (β-ACT) was chosen as the endogenous gene for data normalization because of its high expression level and stability (Table 2).

The real-time quantitative PCR (RT-qPCR) analyses were conducted in duplicate on a QuantStudio 3 Thermocycler (Applied Biosystems™, Foster City, CA, USA), utilizing the Relative Quantification approach. Detection was achieved with the SYBR^®^ Green system (Applied Biosystems, Foster City, CA, USA), and the GoTaq^®^ qPCR Master Mix kit (Promega Corporation, Madison, WI, USA) was used. The PCR followed a specific cycling protocol: an initial denaturation at 95 °C for 2 min, followed by 40 amplification cycles of denaturation at 95 °C for 15 s each, and an annealing and extension phase at 60 °C for 1 min. After amplification, the threshold cycle (Ct) values were normalized employing the ΔCt method, comparing against Ct values derived from the endogenous control gene β-ACT. The quantification of relative gene expression levels was performed employing the 2^−ΔCt^ method as elucidated by Livak and Schmittgen [24].

### 2.4. Statistical Analysis

Analysis of the data was conducted using one-way ANOVA executed through the ExpDes.pt package in R statistical software (version 4.0.4), with the Tukey test applied for mean comparison. A significance level was set at α = 0.05, and each replicate was regarded as an independent experimental unit.

## 3. Results

### 3.1. Growth Performance

The birds that received the LPS challenge without HT supplementation (CON + LPS) showed worse BWG, BW, and FCR, compared to the CON and HT + LPS groups (*p* < 0.001) (Table 3). There were no differences between the treatments on FI (*p* = 0.09).

### 3.2. Relative Organ Weight

Birds challenged with LPS, with or without HT supplementation, had an increase in liver ROW (*p* = 0.001) and in spleen ROW (*p* < 0.0001) compared to CON (Table 4). There were no differences between the treatments for Bursa ROW (*p* = 0.997).

### 3.3. Intestinal Morphometry 

Birds from CON + LPS showed an increase in CD (*p* < 0.001) and decreased the VH:CD ratio of jejunum (*p* = 0.004) when compared to birds in the CON and HT + LPS treatment groups (Table 5). However, there was no difference in VH among the treatment groups (*p* = 0.324). To visualize the effects, images can be found in Figure S1.

### 3.4. mRNA Expression

**Figure 1:**
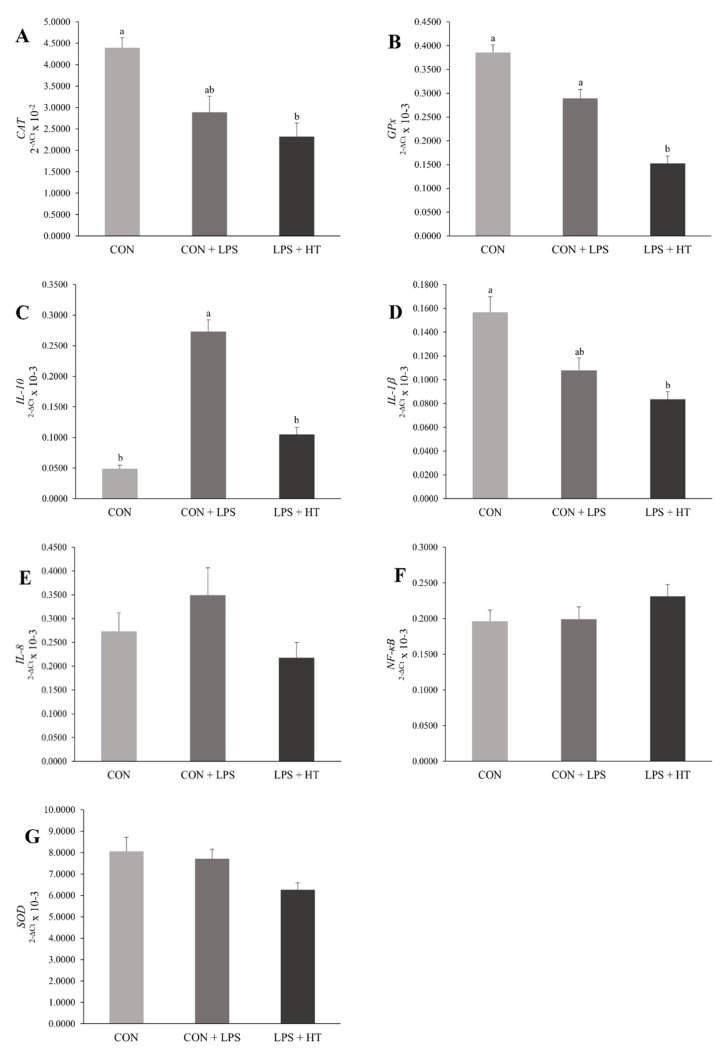
Effects of hydroxytyrosol supplementation on mRNA expression in the jejunum of broilers at 20 days old: (**A**) CAT, *p* value = 0.03; (**B**) GPx, *p* value = 0.0004; (**C**) IL-10, *p* value < 0.0001; (**D**) IL–1β, *p* value = 0.022; (**E**) IL–8, *p* value = 0.213; (**F**) NF–κB, *p* value = 0.633; and (**G**) SOD, *p* value = 0.315. Bars with different letters, along with their corresponding standard errors of the mean, differ from each other by the Tukey test. CON: control diet; CON + LPS: CON with LPS administration; HT + LPS: CON supplemented with 10 mg of hydroxytyrosol/kg of feed with LPS administration. CAT: catalase; GPx: glutathione peroxidase; IL–10: interleukin 10; IL–1β: interleukin 1β; IL–8: interleukin 8; NF–κB: nuclear factor kappa B; SOD: superoxide dismutase.

Birds from the HT + LPS treatment group showed lower gene expression of CAT (*p* = 0.03) and GPx (*p* = 0.0004) when compared to CON (Figure 1A,B). Birds challenged with LPS but without dietary HT showed higher gene expression for IL-10 (*p* < 0.0001) (Figure 1C). Furthermore, birds treated with HT + LPS had lower gene expression of IL-1β compared to CON (*p* = 0.022) (Figure 1D). No significant differences were observed in the gene expression levels of IL-8 (*p* = 0.213), NF-κB (*p* = 0.633), and SOD1 (*p* = 0.315) among the treatment groups (Figure 1E–G).

## 4. Discussion

Gram-negative bacteria have LPS, an endotoxin, as a main component of their cell wall. The release of LPS during bacterial death or rapid growth is associated with the release of pro-inflammatory cytokines and can be harmful to broiler development [25,26]. Previous studies showed that challenges with LPS affected feed intake and weight gain of broiler chickens [3,5,27]. Similarly, in the current study, LPS administration caused a reduction in BWG and BW and increased FCR. However, in the current study, when birds were fed dietary HT, those parameters were not negatively affected when compared to birds in the control treatment. In previous research, supplementation of HT had no influence on broiler growth performance when they were reared under normal conditions [18,19,20]. These previous findings together with the findings of the current study suggest that HT may modulate the inflammatory response, caused by LPS administration, and thus, decrease the diversion of nutrients required to support the immune system due to its anti-inflammatory activity [7], instead of directly promoting broiler growth performance.

The liver, spleen, and bursa of Fabricius play crucial roles in general metabolism and serve as immune organs in broilers. Also, they are LPS target organs, being relevant during the acute-phase immune response [6,28]. The ROW of these organs is a useful parameter to evaluate the immune function of poultry [3]. Several studies have shown that LPS administration caused an increase in the ROW of the liver and spleen, which may be explained by the increase in the production of pro-inflammatory cytokines and recruitment of inflammatory cells to these organs, resulting in its mass growth [6,29,30,31]. We observed that birds that received LPS administration had an increase in ROW of the liver and spleen, regardless of the use or non-use of HT in diets, when compared to birds from the control group. This suggests that dietary HT was not capable of reducing liver and spleen ROW.

The barrier formed by the intestinal mucosa is crucial for maintaining gut balance and acts as the initial defense line against pathogens, facilitated by the digestion and absorption of nutrients [32]. The small intestine is the primary site for nutrient absorption, but it is also a targeted organ for LPS [33,34]. Intestinal mucosa dysfunction is characterized by histological changes such as villus necrosis, reduced VH, and increased CD in the mucosa [35]. An increase in CD and a decrease in VH can impair nutrient absorption, increase the secretion of electrolytes and water in the gastrointestinal tract, and can consequently worsen broiler growth performance [36]. A deeper crypt may imply faster tissue turnover to facilitate the renewal of villi, indicating that the host’s intestinal response mechanism is attempting to compensate for regular sloughing or atrophy of the villus caused by inflammation from pathogens and their toxins [17,37]. Therefore, a high VH:CD ratio is considered a reliable marker of mucosal turnover and is linked to an enhanced capacity for digestion and nutrient absorption [38,39]. The results showed negative effects of LPS administration on intestinal morphometry, evidenced by increasing CD and decreasing VH:CD, consistent with previous findings [2,3,40]. However, dietary HT could attenuate intestinal morphometric damage by decreasing CD and increasing the VH:CD ratio, which consequently could be observed in increased broiler growth performance. These findings could be explained by HT’s role through a direct biological activity in the gastrointestinal tract before being absorbed and also acting on intestinal flora regulation, where HT improves microbial community disturbance and protects intestinal wall integrity [7,41].

LPS initiates the inflammatory process via a Toll-like receptor (TLR4), which is expressed on the cell membrane of leukocytes. After LPS activates TLR4, NF-κB accesses the nucleus and modulates the release of pro-inflammatory cytokines [42,43,44,45]. The interleukin IL-1β plays a pivotal role in regulating the innate immune response in birds [1]. In our study, the LPS challenge did not alter the gene expression of NF-κB and IL-8, while it was observed that the expression of IL-1β was lower in the LPS + HT treatment, compared to CON, but not different from CON + LPS. Therefore, the effects of HT as an anti-inflammatory agent could not be determined in the present study. Moreover, the expression of IL-10 decreased in the LPS + HT treatment when compared to the CON + LPS. IL-10 is known to be a potent anti-inflammatory cytokine that regulates the host immune response to minimize unintended host cell damage during inflammation [46]. The reduction in both interleukins was also observed by Kaiser et al. [47] for vitamin E and Zhang et al. [48] for α-tocopherol succinate supplementations. These results could be attributed to a mechanism of homeostasis in which the appropriate balance of the cytokine network is maintained uniformly for both pro- and anti-inflammatory cytokines.

The administration of LPS not only induces inflammation but also leads to oxidative stress, which is related to inflammatory responses by the release of pro-inflammatory cytokines [26,30,49]. The antioxidant system is composed mainly of SOD, CAT, and GPx, which act as a scavenger of free radicals to decompose hydrogen peroxide (H_2_O_2_) [2,32]. SOD acts on superoxide anion, which is dismutated to H_2_O_2_, and subsequently is catalyzed to H_2_O by CAT, GPx, or peroxiredoxins [50]. Interestingly, we found that dietary HT reduced the expression of GPx and CAT in the jejunum. It suggests that HT may act directly on H_2_O_2_, consequently reducing the need for GPx and CAT to catalyze it into H_2_O. Similar to our finding, Zrelli et al. [13] reported that HT had a protective effect on vascular endothelial cells against the cytotoxic effects of H_2_O_2_. The authors led an investigation to understand the mechanisms involved in this protection and discovered that HT acted through PI3K/Akt and ERK1/2 pathways. These enzymes often play a role in stimulating cell multiplication and enhancing cell survival during oxidative stress [13].

Since the findings showed that HT supplementation decreased antioxidant enzyme expressions, we hypothesized that HT could be acting through another pathway that was not investigated in the present study. The literature shows that HT can modulate the antioxidant response through the nuclear factor erythroid 2-related factor 2 (Nrf2). Nrf2 is one of the major regulators of the antioxidant response and prevents oxidative damage [51]. It can regulate the expression of a diversity of genes related to antioxidant defense and detoxification. Under normal circumstances, Nrf2 is bound to a cytoplasmatic protein called KEAP1, which targets Nrf2 for ubiquitination and subsequent degradation, maintaining low levels of Nrf2 in the cell. On the other hand, under stress conditions, like the LPS challenge in the present study, KEAP1 undergoes a conformational change and thus, does not target Nrf2 for degradation [13,52,53]. Nrf2 activation occurs after its translocation into the nucleus, where it binds to the antioxidant response element (ARE) to promote heme oxygenase 1 (HO-1) and other antioxidant gene expression [13,51,52]. HT has been correlated with the ARE pathway, in which it promotes the activation of various genes encoding ARE, including DNA-repair proteins or phase II detoxifying enzymes [14]. In addition to the activation of PI3K/Akt and ERK1/2 pathways, HT induces the expression of HO-1, a downstream phase II detoxifying enzyme, after Nrf2 activation [13]. HO-1 reduces intracellular ROS, therefore, contributing to the cytoprotective effects of HT against oxidative stress [13]. According to Satta et al. [51], the Nrf2/HO-1 signaling pathway has a pivotal role in decreasing oxidative stress levels in the organism. 

In the present study, HT acted by reducing the oxidative stress in the broiler’s jejunum. It could be related mainly to HT’s direct role against the cytotoxic effects of H_2_O_2_ as previously discussed. Moreover, it might have induced an increase in HO-1 gene expression, which acts to reduce intracellular ROS; however, it was not investigated.

Briefly, HT has an important role in the antioxidant response, in which the proper mechanisms need to be further investigated to understand HT’s antioxidant capacity either directly or indirectly.

## 5. Conclusions

The supplementation of 10 mg of HT/kg of feed attenuates the reduced growth performance and the jejunal mucosa damage caused by LPS challenge by increasing villus height to crypt depth ratio and improving the oxidative response of broiler chickens. More studies should be carried out to better elucidate if HT has anti-inflammatory effects on broilers and to understand the proper mechanisms by which HT can enhance the oxidative responses of broiler chickens.

## Figures and Tables

**Table 1:** Components and nutritional calculation of the basal diets (as-fed basis).

Ingredient	8 to 20 Days, %
Corn, 7.88%	50.38
Soybean meal, 45.0%	41.12
Soybean oil	4.58
Dicalcium phosphate	1.67
Limestone	0.84
Salt	0.52
DL-Methionine, 99%	0.32
L-Lysine, 78%	0.15
L-Threonine, 98.5%	0.05
Choline chloride, 60%	0.10
Phytase	0.01
Antioxidant (BHT) ^3^	0.01
Mineral Premix ^1^	0.13
Vitamin Premix ^2^	0.13
Calculated nutritional composition ^4^	
Crude Protein, %	22.3
Metabolizable Energy, kcal/kg	3000
Calcium, %	0.980
Available Phosphorus, %	0.480
Sodium, %	0.220
Digestible Lysine, %	1.240
Digestible Methionine, %	0.470
Digestible Methionine + Cysteine, %	0.929
Digestible Threonine, %	0.810
Digestible Tryptophan, %	0.267

^1^ Composition per kg of product: Manganese, 58.36 g; Iron, 41.68 g; Zinc, 54.21 g; Copper, 8.31 g; Iodine, 0.84 g; Selenium, 0.25 g. ^2^ Composition per kg of product: Vitamin A, 9,638,000 IU; Vitamin D3, 2,410,000 IU; Vitamin E, 36,100 IU; Vitamin B1, 2.60 g; Vitamin B2, 6.45 g; Vitamin B6, 3.61 g; Vitamin B12, 15.9 mg; Vitamin K3, 1.94 g; Pantothenic Acid, 12.95 g; Nicotinic Acid, 39.20 g; Folic Acid, 0.90 g; Biotin, 89.80 mg. ^3^ Antioxidant Butylhydroxytoluene. ^4^ Calculated following Rostagno et al. [21].

**Table 2:** Sequences of primers utilized in gene expression analysis.

Gene	GenBank	Sequence
*NF-κB*	NM_205129.1	F: 5′-GTGTGAAGAAACGGGAACTG-3′
		R: 5′-GGCACGCTTGTCATAGATGG-3′
*CAT*	NM_001031215.2	F: 5′-ACTGCAAGGCGAAAGTGTTT-3′
		R: 5′-GGCTATGGATGAAGGATGGA-3′
*GPX*	NM_001277853.2	F: 5′-GACCAACCCGCAGTACATCA-3′
		R: 5′-GAGGTGCGGGCTTTCCTTTA-3′
*SOD1*	NM_205064.1	F: 5′-AGGGGGTCATCCACTTCC-3′
		R: 5′-CCCATTTGTGTTGTCTCCAA-3′
*IL-10*	NM_001004414.2	F: 5′-CATGCTGCTGGGCCTGAA-3′
		R: 5′-CGTCTCCTTGATCTGCTTGATG-3′
*IL-1β*	NM_204524.1	F: 5′-GCTCTACATGTCGTGTGTGATGAG-3′
		R: 5′-TGTCGATGTCCCGCATGA-3′
*IL-8*	HM179639.1	F: 5′-GGCTTGCTAGGGGAAATGA-3′
		R: 5′-AGCTGACTCTGACTAGGAAACTGT-3′
*β-ACT*	NM_205518.1	F: 5′-TGCTGTGTTCCCATCTATCG-3′
		R: 5′-TTGGTGACAATACCGTGTTCA-3′

**Table 3:** Feed intake (FI), body weight gain (BWG), body weight (BW), and feed conversion ratio (FCR) of broilers from 8 to 20 days.

	CON	CON + LPS	HT + LPS	*p* Value	SEM
FI, kg/bird	0.934	0.926	0.910	0.09	0.005
BWG, kg/bird	0.741 ^a^	0.704 ^b^	0.736 ^a^	<0.001	0.005
BW, kg/bird	0.973 ^a^	0.932 ^b^	0.967 ^a^	<0.001	0.005
FCR, kg/kg	1.262 ^a^	1.317 ^b^	1.238 ^a^	<0.001	0.010

^a,b^ Letters differing within the same row indicate significant differences according to the Tukey Test (α = 0.05). SEM: standard error of the mean.

**Table 4:** Relative organ weight (ROW) of liver, spleen, and Bursa of broilers at 20 days old.

	CON	CON + LPS	HT + LPS	*p* Value	SEM
Liver, %	2.494 ^b^	3.038 ^a^	2.853 ^a^	0.001	0.067
Spleen, %	0.099 ^b^	0.167 ^a^	0.150 ^a^	<0.0001	0.007
Bursa, %	0.214	0.213	0.213	0.997	0.009

^a,b^ Letters differing within the same row indicate significant differences according to the Tukey Test (α = 0.05). SEM: standard error of the mean.

**Table 5:** Villus height (VH), crypt depth (CD), and villus: crypt ratio (VH:CD) of jejunum from broiler chickens at 20 days old.

	CON	CON + LPS	HT + LPS	*p* Value	SEM
VH, µm	2100.85	1972.55	2108.57	0.324	40.91
CD, µm	477.77 ^a^	582.77 ^b^	448.81 ^a^	<0.001	15.28
VH:CD	4.453 ^a^	3.417 ^b^	4.879 ^a^	0.004	0.198

^a,b^ Letters differing within the same row indicate significant differences according to the Tukey Test (α = 0.05). SEM: standard error of the mean.

## Data Availability

Data are contained within the article and Supplementary Materials.

## Supplementary Materials

The following supporting information can be downloaded at: https://www.mdpi.com/article/10.3390/ani14060871/s1, Figure S1: Images of jejunum to illustrate the effects of hydroxytyrosol supplementation on the morphology, collected from broiler chickens at 20 days old, challenged or not by LPS.
