# Supplementary material for: Dietary Hydroxytyrosol Supplementation on Growth Performance, Gut Morphometry, and Oxidative and Inflammatory Status in LPS-Challenged Broilers
Source: Animals (Basel). 2024 Mar 12;14(6):871. doi: 10.3390/ani14060871 (PMC10967364; doi:10.3390/ani14060871)
Supplement: Supplementary file 1 [file animals-14-00871-s001.zip › animals-2872429-supplementary.pdf]

Figure S1. Images of jejunum to illustrate the effects of hydroxytyrosol supplementation on the morphology, collected from broiler chickens at 20 days old, challenged or not by LPS.

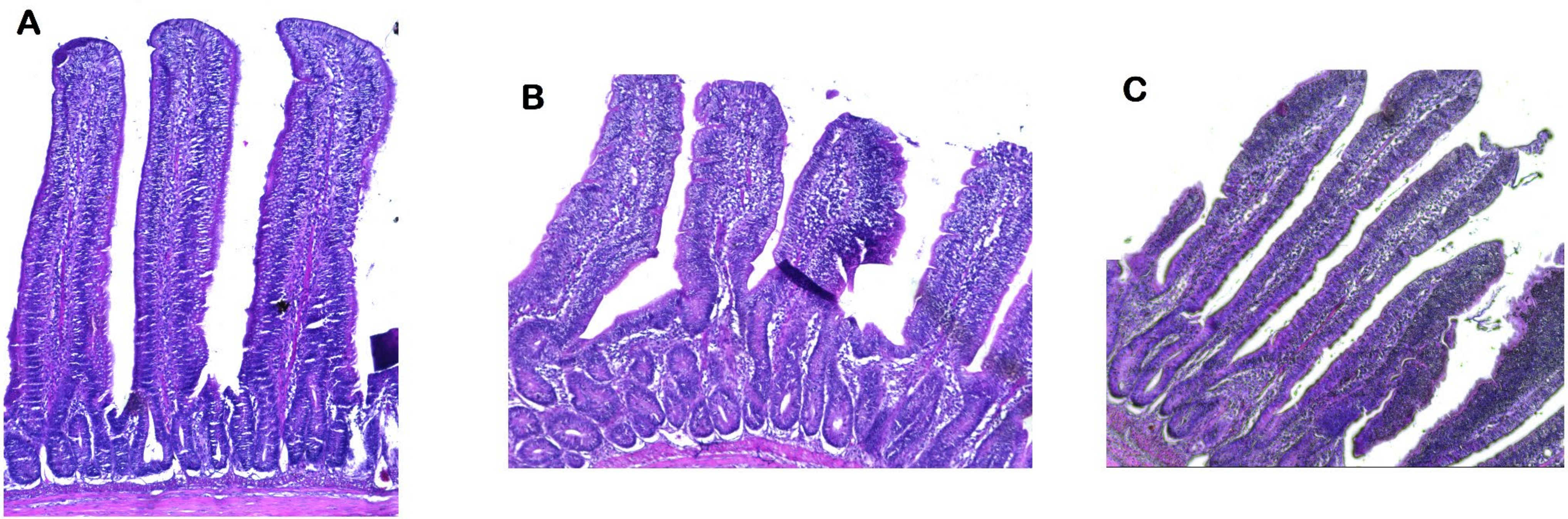

A: control diet without LPS administration; B: control diet with LPS administration; C: control diet supplemented with 10 mg of hydroxytyrosol/kg of feed with LPS administration.
